# The Increase of Flavonoids in Pericarpium Citri Reticulatae (PCR) Induced by Fungi Promotes the Increase of Antioxidant Activity

**DOI:** 10.1155/2018/2506037

**Published:** 2018-12-02

**Authors:** Fu Wang, Lin Chen, Feng-qing Li, Su-juan Liu, Hong-ping Chen, You-ping Liu

**Affiliations:** Department of Pharmacy, Chengdu University of TCM, Chengdu, Sichuan, China

## Abstract

There are thousands of traditional Chinese medicines in China, and they all have to be stored for a period of time for the reason of market price or other factors. But some traditional Chinese medicines especially need to be stored longer, and the clinical efficacy will be better. The cause and mechanism of this phenomenon have attracted much attention in recent years. In this study, we analyze the reason of “the longer storage period the better” of Pericarpium Citri Reticulatae (PCR); method of microscopic and molecular identification was used to identify the fungi separated and purified from PCR. The HPLC and UV spectrophotometry methods were used to determine the contents of flavonoids in PCR. The isolated fungi were inoculated into sterile samples to screen the fungi closely related to the change of flavonoids. The results indicate that the strain of* Aspergillus niger *could obviously promote the contents of flavonoids, and it could also increase the antioxidant effect of PCR. In conclusion, this study explains the reason of “the longer storage period the better” of PCR from the perspective of microbe, proving the beneficial effect of microorganism on the surface of PCR.

## 1. Introduction

Tangerine is distributed throughout the world and there are many varieties of variations; the fruit is sweet and sour and is loved by the people all over the world. In 2014, China's citrus cultivation area was 3432 million hectares, with an annual output of 29.44 million tons, and its cultivated area and yield were the world's largest. Pericarpium Citri Reticulatae (PCR) is a traditional Chinese medicine from the dried and ripe peel of* Citrus reticulata *Blanco and its cultivated varieties [1]; it has been widely used in traditional Chinese medicine prescription for about 2000 years up to now and has the function of eliminating dampness and phlegm. The theory of traditional Chinese medicine believes that “the longer storage period the better” of PCR; therefore, we can find different years of PCR in the market, and the longer the storage, the higher the price; more than 10-year-old PCR is also known as “soft gold.” Therefore, it is of great practical significance to explore the causes of “the longer storage period the better” of PCR.

The reason “the longer storage period the better” of PCR had been studied by many researchers [2–4]; it was generally believed that the accumulation of active substances may occur during the long-term storage, so as to achieve the goal of increasing efficacy. According to the literatures [5–7], the effective substances of PCR were mainly volatile oil and flavonoids; the contents of volatile oil would reduce in the process of storage, but the contents of flavonoids increase. We all know that volatile oil is volatile and the content will decrease continuously, but the reason for the increase of flavonoids is not clear, which cannot explain the reason “the longer storage period the better” of PCR. Other researchers reported that the microbe of the plants could promote the accumulation of the active ingredients [8, 9]. By the way of thinking, this paper puts forward assumption: the process of storage of PCR may be accompanied by the metabolic transformation of fungi, though the fungi cannot be detected by the naked eye, but with the accumulation of time and the long-term metabolic activities of fungi, the contents of flavonoids can be changed.

**Figure 1:**
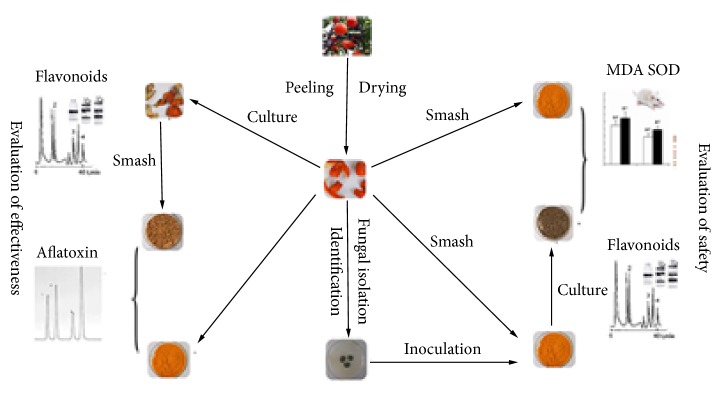
The framework of the study on the reason of “the longer storage period the better” of PCR.

In the existing literature, the research methods of the reasons “the longer storage period the better” often used different storage years samples of PCR (1~40 years) under the natural state [10, 11]; however, it is difficult to collect samples in different years, and the authenticity of the samples in different years of the market is difficult to prove, which makes it more difficult to explore the reasons for “the longer storage period the better” of PCR. In this study, we put forward the following idea: the metabolic transformation of the microorganism on the surface of PCR may be closely related to the cause of the “the longer storage period the better”; the effects of microorganisms on the main active components and pharmacological effects were studied by using the method of constant temperature and humidity accelerated culture (Figure 1). Firstly, the isolation, purification, and identification of the fungi on the surface of PCR were carried out to figure out the growth of fungal groups in different batch samples. Secondly, the contents of 4 flavonoids and total flavonoids in samples before and after accelerated culture were determined to understand the changes of flavonoids. Thirdly, the isolated fungi were then inoculated to the sterile PCR to screen the fungal strains that caused the changes of flavonoids. In the end, a comparative experiment on antioxidant capacity of the sample that inoculated fungi and the samples stored for 1 year and 3 years was carried out to understand the difference between them. Through the above experiments, we hope to provide a scientific explanation for “the longer storage period the better” of PCR.

## 2. Materials and Methods

### 2.1. Sample Information

The samples used in the experiment were identified by Professor Yan Zhuyun, Chengdu University of Traditional Chinese Medicine (Table 1). They were the dried and ripe peel of* Citrus reticulata Blanco* and its cultivated varieties.

### 2.2. Instruments and Reagents

They were as follows: Retsch MM400 Ball Mill(Laichi co., Germany); PTC200PCR(BIO-Rad, American); GelDox XR gel imaging system (BIO-Rad, American); DYY-8C Electrophoretic apparatus (Beijing Liuyi Instrument, China); BI3730XL sequenator (Applied Bio-systems, American); KRQ-300P manual climatic box (ChongQing YinBe Test Instrument, China); Plant DNA Extraction Kit (Tiangen Biotech Co., China); 2×Taq PCR MasterMix (Tiangen Biotech Co., China); primer was compounded by Sangon Co., China. Shimadzu; LC-20AT HPLC (Shimadzu Co., Japan); DM4000M microscope (Leica, Germany); Agilent 8453 ultraviolet and visible spectrophotometer (Agilent Technologies Co., American); hesperidin (MUST-12041206); narirutin (MUST-14081915); hesperetin (MUST-14100914); naringenin (MUST-14112310), four reference substance were bought from Chengdu Mansite biological technology co. LTD, purity>98.0%. The other reagents were analytically pure.

### 2.3. Isolation and Identification of Growth Fungi on the Surface of PCR

#### 2.3.1. Isolation Method [12]

The methods for separating the growing fungi on the surface of PCR are as follows: Take 5.0 g from each batch sample of PCR(PCR1~PCR8), put it in the 50 mL sterile centrifuge, add it to 30 mL 0.1% Tween-20, agitate it violently for 3 min, filter it with disposable syringe full of sterile cotton, collect filtrate, 5000 r·min^−1^centrifuge in 10 min, discard the supernatant, resuspend the sediment in 300 *μ*L 40% sterile glycerinum, dilute it to 1×10^−4^ bacterium liquid, respectively, take 100 *μ*L to coat on PDA plate (chloromycetin concentration 0.1 g·L^−1^), and incubate it for 3 days in constant temperature 30°C. After observation of medium growth colonies, transfer them to the new PDA plate by inoculating loop (chloromycetin concentration 0.1 g·L^−1^); continue to cultivate, inoculate, and transfer to the PDA test tube after purification for strains species identification.

#### 2.3.2. Microscopic Identification

In the ultraclean workbench, put the coverslip on PDA medium inclined, inoculate the purified strains with inoculating loop, and cultivate them for 3 days in the incubator in constant temperature 30°C. Shave the medium lower end of the coverslip by blade, and drip a drop of distilled water on the central of the glass slide. Then put on the coverslip, and observe and take pictures by low power lens and high power lens under a microscope [13].

#### 2.3.3. Molecular Identification

Extract DNA from samples by DNA extraction kits; amplify ITS sequence by universal primer ITS1/ITS4 of DNA barcode. Reaction conditions and amplification procedure refer to the research of C.Arif [14]. Bidirectional DNA sequence after PCR products has been purified. Proofread and splice the sequence map which comes from data processing by soft CodonCodeAligner V5.0.2(CodonCode Co., USA); remove the primer and inferior quality sequence. Construct neighbor-joining phylogenetic tree by soft Maga 5.1; check the approval rating of each branch by Bootstrap. Identify and analyze by similarity search method.

### 2.4. Detection of Aflatoxin in the Samples of PCR

#### 2.4.1. Chromatographic Conditions

The method refers to the Chinese Pharmacopoeia 2015 edition. The chromatographic column is Sunfire C_15_(4.6×250 mm, 5 *μ*m). The mobile phase is methanol acetonitrile and water (40:18:42). Flow rate is 0.8 ml/min. Derivatization solution is 0.05% iodine solution. The excitation wavelength is 360 nm, and the emission wavelength is 335 nm. Injection volume is 10.0 *μ*L.

#### 2.4.2. Preparation of Standard Solution

Take aflatoxins G2(0.59 *μ*g/ml), G1(1.18 *μ*g/ml), B2(0.35 *μ*g/ml), and B1(1.04 *μ*g/ml)1 ml, weigh them precisely, put them in 50 ml measuring flask, dilute them to scale with methanol, and shake them as a reverse liquid I. Measure accurately liquid I 2 ml to 10 ml measuring flask, dilute it to scale with methanol, shake it as a reverse liquid II, then take 1ml liquid II to the 10 ml measuring flask, dilute it to the scale with methanol, and shake and obtain the standard solution.

#### 2.4.3. Preparation of Sample Solution

Take 10 g powder of the sample of PCR (coarse powder), weigh it precisely, add 3 g sodium chloride in the homogenized bottle and 70% methanol 75 ml, with high-speed stirring for 2 minutes (stirring speed greater than 11000 RPM), and then centrifuge for 5 minutes (centrifugal speed 2500 RPM). Take the supernatant, 15 ml precisely, and put it into 50 ml volumetric flask, dilute it with water to the scale, shake and filter it with microporous membrane (0.45 mm), take the filtrate 20.0 ml, through the immunoaffinity column, flow rate 3 ml per minute, 20 ml water elution, discard eluent, put the air into the column, squeeze water out of the column, and 10 ml methanol elution, collect the eluent into a 2 ml volumetric flask, and dilute it with methanol to the scale, and then shake it and obtain the sample solution.

#### 2.4.4. Determination of Aflatoxin in Samples

Prepare the sample solution as mentioned above; determine aflatoxin content in the selected chromatographic conditions, each parallel batch sample determined 3 times; measurements are averaged.

### 2.5. The Culture Method of Samples

Take 100.0 g from each batch sample, put it in the sterile culture box, and then cultivate it for about 5 days in the artificial climate box (T=30°C, RH=95%).

### 2.6. Determination of the Content of 4 Flavonoids in the Samples before and after Culture

#### 2.6.1. Chromatographic Conditions

Refer to the method which our group established [15], as follows: chromatographic column is Hypersil BDS C18(4.6×200 mm, 5 *μ*m); moving phase: 0.05% phosphoric acid water and acetonitrile, follow the gradient elution program: time: 0~5 min, acetonitrile: 20%~25%; time: 5~10 min, acetonitrile: 25%~30%; time: 10~20 min, acetonitrile: 30%~50%; time: 20~30 min, acetonitrile: 50%~60%; time: 30~35 min, acetonitrile: 60%~90%; time: 35~40 min, acetonitrile: 90%~100%; column temperature: 30°C; flow velocity 0.7 mL·min^−1^; determine wavelength: 283 nm and 335 nm; injection volume: 5.0 *μ*L.

#### 2.6.2. Preparation of Sample Solution

Take 0.2 g powder (coarse powder, before and after accelerated culture), weigh it precisely, add methanol 25 mL, weigh it, reflux for 1 hour in the 75°C water bath, weigh it once again after chilling, make up weight with solvent, filter it, take further filtrate to filter with 0.45 *μ*m microporous membrane, and then obtain the sample solution.

#### 2.6.3. Preparation of Standard Solution

Weigh narirutin, hesperidin, hesperetin, and naringenin appropriately and precisely, add methanol to constant volume 25 mL, and prepare mixed standard solutions of 0.0544 mg·mL^−1^of narirutin, 0.1348 mg·mL^−1^ of hesperidin, 0.1128 mg·mL^−1^ of hesperetin, and 0.0540 mg·mL^−1^ of naringenin.

#### 2.6.4. Determination of 4 Flavonoids in the Samples

Take 0.2 g powder (coarse powder, before and after accelerated culture), weigh it precisely, prepare sample solution as mentioned above, determine 4 flavonoids contents in the selected chromatographic conditions, each parallel batch sample determined 3 times, and measurements are averaged.

### 2.7. Determination of Total Flavonoids in the Samples before and after Culture [16]

#### 2.7.1. Preparation of Standard Solution

Take some hesperidin standard substance, weigh it precisely, add methanol to constant volume 10 mL, and then prepare standard solutions of 0.36 mg·mL^−1^ of hesperidin.

#### 2.7.2. Preparation of Sample Solution

Take 0.2 g powder (coarse powder, before and after accelerated culture), weigh it precisely, add methanol 25 mL, weigh it again, reflux for 1 hour in the 75°C water bath, weigh it once again after chilling, make up weight with solvent, and filter and obtain the sample solution.

#### 2.7.3. Drawing of Standard Curve

Take hesperidin standard solution 0.25, 0.50, 1.0, 1.2, 1.5, and 2.0 mL into 25 mL volumetric flask, respectively, constant volume to scale with methanol. Concentration of hesperidin solution is abscissa(*X*), absorbance value is coordinate(*Y*), for linear regression analysis; obtain the regression equation* Y*=0.0297*X*+0.0257,* R*^*2*^=0.9996.

#### 2.7.4. Determination of Total Flavonoid in the Samples

Take 0.2 g powder (coarse powder, before and after accelerated culture), weigh it precisely, prepare sample solutions as the above-mentioned method. Take 0.5 mL precisely into 25 mL volumetric flask, dilute it to the scale with methanol, determine the absorbance in 283 nm, each parallel batch sample determined 3 times, measurements are averaged, and then calculate the general flavonoid contents in each batch of PCR.

### 2.8. Sterilization, Reverse Inoculation, and Contents Determination of PCR

#### 2.8.1. Sterilization and Reverse Inoculation

Take 15 g sample of PCR2 to the bottom of culture dish, sterilize it for 30 min under ultraviolet light, turn it over, and continue for 30 min. Divide the sample into blank group and test group (*Aspergillus flavus, Aspergillus niger, Penicillium citrinum, Penicillium milmonillium*,* and Penicillium common*). Test group: add standard spore suspension 1mL to each culture dish, and number the samples. Blank group: add sterile water 1 mL to each culture dish, and number the samples. Two groups of samples are cultivated for about 5 days (T=30°C, RH=95%), each sample parallel 3 times.

#### 2.8.2. Contents Determination

Determine contents of 4 flavonoids and total flavonoids with the above-mentioned methods.

### 2.9. Comparison of Antioxidant Effects of Different Storage Years (1 Year and 3 Years) with the Sample Which Inoculated* Aspergillus niger*

#### 2.9.1. Experimental Method

Seventy-two healthy KM males were adapted to be raised for 3 days. According to their weight, they were randomly divided into 6 groups, namely, blank group, CCl_4_ group, VC (1 g·L^−1^) group, S1 group (inoculate* Aspergillus niger*), 1 year group (PCR5), and 3 years group (PCR6), 12 for each group. For the drug, it was 1 time per day, 7 days for continuous irrigation, 0.2 ml/10 g for stomach volume, and an equal amount of distilled water for blank group and model group. After 1 h of irrigation, in addition to the blank group, the other experimental groups were treated with 0.2 ml of 0.2% CCl_4_, and the blank group was replaced with equal vegetable oil, and blood was taken from orbit after 16 hours. 3500 r·min^−1^, 10 min 4°C centrifugal separation of serum, repackaging, -80°C keep spare. After the serum was taken, the mice were dissected and the liver was prepared to prepare the liver homogenate, and the SOD and MDA activity were measured in the hepatic homoserous fluid.

#### 2.9.2. Data Analysis

Statistical software SPSS 21.0 was adopted to carry out the data statistics. The measurement data conforms to the normal distribution, and the one-way ANOVA analysis was adopted; nonparametric test was used to analyze the nonconforming.

## 3. Results and Discussion

### 3.1. Microscopic Identification

**Figure 2:**
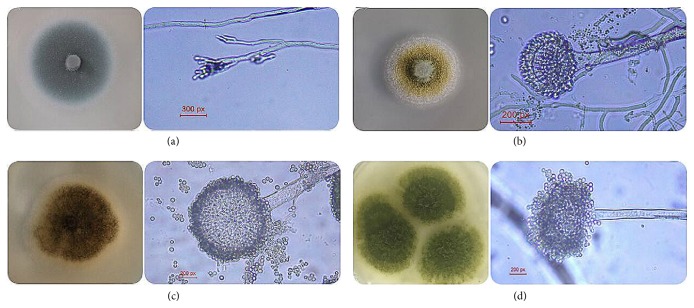
The shapes and microscopic characters of fungal separated from PCR. (a)* Penicillium citrinum*; (b)* Aspergillus flavus*; (c)* Aspergillus niger*; (d)* Penicillium minioluteum*.

To understand the fungal groups which grow on the surface of PCR, the separation and microscopic identification of 8 batches of samples were carried out in this experiment. The results have shown that 25 strains were isolated, belonging to 2 genera and 5 species, all belonging to* Penicillium *genus* and Aspergillus*, respectively, for* Penicillium common*,* Penicillium milmonillium, Penicillium citrinum, Aspergillus flavus*, and* Aspergillus niger*; dominant fungi was* Aspergillus niger*. The morphology and micrograph of some strains are shown in Figure 2. The results have shown that (Table 2) the fungal growth on the surface of PCR was relatively fixed, mainly for the* Penicillium *genus* and Aspergillus*;* Aspergillus niger *could only be isolated in all the samples;* Penicillium citrinum, Penicillium milmonillium,* and* Penicillium common* could be isolated in individual samples.

### 3.2. Molecular Identification

**Figure 3:**
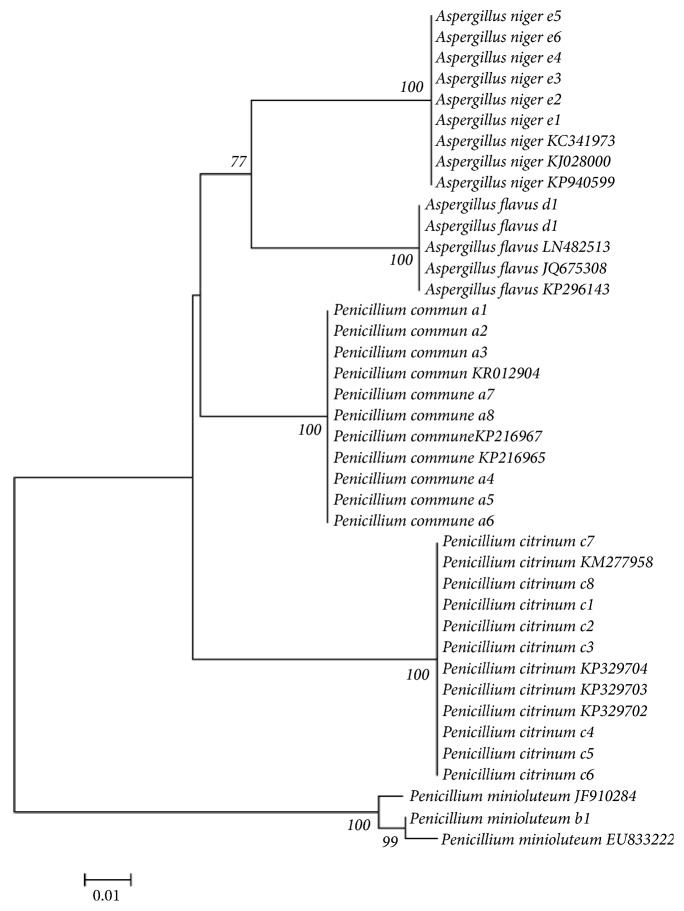
The NJ tree constructed based on the K2P of the ITS sequence. a1~a8(*P. commune*); b1(*P. minioluteum*); c1~c8(*P. citrinum*); d1~d2(*A. flavus*); e1~e6(*A. niger*); KC341973, KJ028000, KP940599, LN482513, JQ675308, KP296143, KR012904, KP216967, KP216956, KM277958, KP329704, KP329703, KP329702, JF910284, and EU833222 downloaded from NCBI database.

Because P.* commune*, P.* citrinum*, and P.* minioluteum* have similar microscopic structure, PCR amplification and sequencing experiments were used to identify all the 25 strains separated from samples. The results indicated that they were all belonging to* Penicillium *genus* and Aspergillus*. Neighbor-joining phylogenetic tree was constructed to distinguish each fungus. P.* commune*, P.* citrinum*, A.* niger*, P.* minioluteum*, and A.* flavus* were, respectively, clustered with the corresponding sequences downloaded from NCBI (Figure 3). The results of molecular identification were in agreement with microscopic identification, which verify the accuracy of microscopical identification. The results laid solid foundation for the follow-up experiments.

### 3.3. Aflatoxin Detection

**Figure 4:**
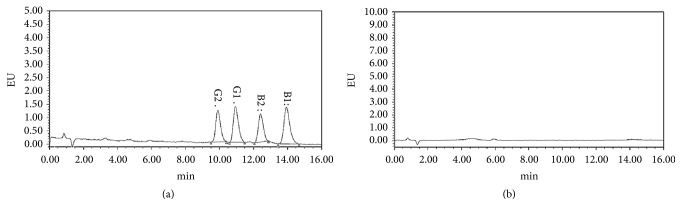
The chromatogram of aflatoxin G2, G1 and B2, B1. (a) The chromatogram of aflatoxin G2, G1 and B2, B1. (b) The chromatogram of the sample of PCR1.

In this study,* Aspergillus flavus *was isolated from the samples of PCR4 and PCR6;* Aspergillus flavus *had attracted much attention because of aflatoxins. At the same time, the Chinese pharmacopoeia of 2015 edition stipulated the limits of aflatoxins. Therefore, this experiment detected aflatoxin in all samples of PCR in consideration of safety reasons. It was known from the chromatogram (Figure 4) that the separation degrees of aflatoxins G2, G1, B2, and B1 were all good, but the G2, G1, B2, and B1 of aflatoxin were not detected in the sample chromatogram. The results have shown that there were no aflatoxins G2, G1, B2, and B1 in all the samples. So we can say that the samples of PCR will grow fungi in the process of storage, even* Aspergillus flavus*, such as PCR4 and PCR6, but it does not mean the sample will contain aflatoxin. It is suggested that the storage of PCR under suitable conditions can be absolutely safe.

### 3.4. The Changes of Flavonoids before and after Culture of the Samples of PCR

**Figure 5:**
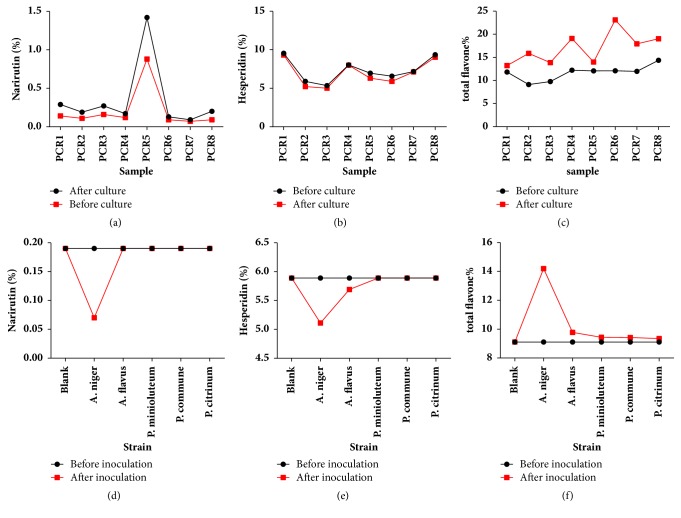
The changes of flavonoids of the samples of PCR. (a)~(c) The changes of flavonoids before and after culture. (d)~(f) The changes of flavonoids before and after inoculation.

The accelerated cultivation of temperature and humidity (T=30°C, RH=95%) in this experiment was the best condition for microorganism growth and could accelerate the growth and metabolism of microorganisms. Thus, the relationship between microorganism and flavonoids of PCR could be studied rapidly. It was known by HPLC measurement (Table 3) that the contents of narirutin and hesperidin decreased, while the contents of their transformation product, hesperetin and naringenin, increased. At the same time, total flavonoids increased significantly after accelerated culture, and the change trend is shown in Figures 5(a)–5(c). The results have shown that the metabolic activity of the microorganism could increase the content of total flavonoids and promote the transformation of flavonoids of PCR.

At present, plenty of researches had been carried out to verify the reason of “the longer storage period the better” of PCR; they all proved that the content of active components of PCR was changed in storage, but how the effective components change is still unknown. Our team put forward the hypothesis that the change of active ingredients will occur by long-term microbial metabolism on the basis of previous studies. According to the results of contents determination, the total flavonoids increased and the components were transformed between them after accelerated culture; these results are consistent with the flavonoids increasing which the literature had reported [17]. So we preliminarily confirmed that the metabolic activity of the microorganism could significantly increase the content of flavonoids and promote the transformation of flavonoids of PCR.

### 3.5. Screening of Fungal Strains That Lead to the Change of Flavonoids

The experiment (3.4) had confirmed that microorganism could increase the contents of flavonoids and promote the transformation of flavonoids of PCR, but whether one or several microorganisms cause the effect was unknown. So we inoculated the fungus isolated from the surface of PCR back into the sterile PCR to screen the strains that cause the changes of flavonoids. The results of HPLC (Table 4) have shown that the contents of flavonoids in the blank group were unchanged, indicating that the effect of sterilization was good. And all the five fungi caused the mold of PCR in the test group, but only the strain of A.* niger* could significantly enhance the total flavonoids and decrease the content of narirutin and hesperidin in PCR compared to other strains; the change trends are shown in Figures 5(d)–5(f). It was reported that* Aspergillus niger* has the ability to convert flavonoids; the results of the study were in agreement with the literature [18–21]. At the same time, the microbial transformation products were detected from the sample: hesperetin and naringenin; these two biotransformation compounds have phenolic hydroxyl groups at the 5,7 position in the A ring, which have direct impact on the increase of the antioxidant properties.

### 3.6. Comparison of Antioxidative Effects between Different Storage Years of PCR and the Sample That Inoculated* Aspergillus niger*

The experiment (3.5) had confirmed that the strain of* Aspergillus niger *could obviously cause the increase the content of flavonoids and promote the transformation of flavonoids of PCR. But what about the pharmacological effect of the sample that inoculated* Aspergillus niger*? So the pharmacodynamic effects of different storage years of PCR and the sample that inoculated* Aspergillus niger* were further compared. Compared with the CCl_4_ group, the content of SOD in the blank group increased significantly, and the MDA content decreased significantly, indicating that the liver injury model was successfully replicated (Table 5). Compared with the CCl_4_ group, the activity of SOD in the group S1 and the 3 years PCR group increased significantly and the activity of MDA decreased significantly (P<0.05), which means both of them had antioxidative effect. As can be seen from experimental data, the antioxidant capacities of the group S1 and 3 years PCR group were similar, but the antioxidant capacity of the group S1 was higher than the 1 year PCR group. So we could draw a conclusion: the strain of* Aspergillus niger* screened from the experiment had the function of transforming flavonoids from PCR and could promote the increase of flavonoids and enhance their antioxidant capacity.

The results verified that* Aspergillus niger* was involved in the transformation of flavonoids, which promoted the addition of flavonoids and the enhancement of pharmacological effect, thus confirming the reason “the longer storage period the better” of PCR. At the same time, the results suggest that PCR will grow different populations of fungi during storage due to the environmental and storage conditions or other factors, even including* Aspergillus flavus, *but it does not mean that there are aflatoxins in the samples; we should not have a phobia about the fungi on the surface of PCR. Instead, it is because of the growth and metabolism of the fungi that promote the accumulation of active ingredients and the increase of pharmacodynamic effect. And this is also the real reason about “the longer storage period the better” of PCR.

## 4. Conclusion

In this paper, the effects of microbes on flavonoids were discussed for the first time to explain the reason of “the longer storage period the better” of PCR. The fungi growing on the surface of PCR, especially the strain of* Aspergillus niger*, could increase the contents of flavonoids and promote the transformation of flavonoids, and its transformation products have abundant hydroxyl groups in the A ring; therefore, its antioxidant capacity is greatly enhanced. The experimental results provide another evidence for the reason of “the longer period the better” of PCR, which is not only related to volatile aroma components reported, but also related to the changes of flavonoids. At the same time, the strain of* Aspergillus niger*, isolated from the surface of PCR, will also have great application prospect in the high efficiency extraction of flavonoids, the conversion of active ingredients, and the improvement of the quality of PCR.

## Figures and Tables

**Table 1:** The smples used in the study.

Number of samples	Species names	Latin name	Source	Storage time
PCR1	Dahongpao	*Citrus reticulate *“Dahongpao”	Sichuan Jintang	Two years
PCR2	Dahongpao	*Citrus reticulata* Dahongpao'	Sichuan Pujiang	Two years
PCR3	Dahongpao	*Citrus reticulata* Dahongpao'	Sichuan Qingbaijiang	Two years
PCR4	Dahongpao	*Citrus reticulata* Dahongpao'	Sichuan Meishan	Five years
PCR5	Chachi	*Citrus reticulata* “Chachi”	Guangdong Xinhui	One year
PCR6	Dahongpao	*Citrus reticulata* Dahongpao'	Sichuan Pujiang	Three years
PCR7	Dahongpao	*Citrus reticulate *Dahongpao'	Sichuan Qingbaijiang	Two years
PCR8	Dahongpao	*Citrus reticulata* Dahongpao'	Sichuan Renshou	Four years

**Table 2:** The statistics of fungi separated from PCR.

Number of samples	Source	The number of fungi	strains				
			*Penicillium common*	*Penicillium citrinum*	*Aspergillus flavus*	*Aspergillus niger*	*Penicillium minioluteum*
PCR1	Sichuan Jintang	2	-	-	-	+	+
PCR2	Sichuan Pujiang	3	+	+	-	+	-
PCR3	Sichuan Qingbaijiang	3	+	+	-	+	-
PCR4	Sichuan Meishan	4	+	+	+	+	-
PCR5	Guangdong Xinhui	4	+	+	-	+	+
PCR6	Sichuan Pujiang	3	+	-	+	+	-
PCR7	Sichuan Qingbaijiang	3	+	+	-	+	-
PCR8	Sichuan Renshou	3	-	+	-	+	+
Total		25	6	6	2	8	3

The “+” represents having corresponding fungi. The “-” represents having no corresponding fungi.

**Table 3:** The changes of flavonoids before and after culture of the samples of PCR.

NO.	Narirutin %		Hesperidin %		Hesperetin %		Naringenin %		total flavone%	
	Before culture	After culture	Before culture	After culture	Before culture	After culture	Before culture	After culture	Before culture	After culture
PCR1	0.29	0.14	9.54	9.32	0	0.22	0	0.14	11.83	13.22
PCR2	0.19	0.11	5.89	5.22	0	0.59	0	0.07	9.11	15.87
PCR3	0.27	0.16	5.35	5.01	0	0.34	0	0.11	9.77	13.86
PCR4	0.17	0.12	8.03	7.99	0	0.14	0	0.06	12.21	19.09
PCR5	1.42	0.88	6.96	6.30	0	0.58	0	0.29	12.08	13.99
PCR6	0.13	0.09	6.57	5.89	0	0.68	0	0.06	12.10	23.11
PCR7	0.09	0.07	7.17	7.12	0	0.11	0	0.06	11.97	17.92
PCR8	0.20	0.09	9.35	9.03	0	0.28	0	0.11	14.37	19.03

**Table 4:** The effect of different fungi on the contents of flavonoids.

Group	Strains	Narirutin %		Hesperidin %		Hesperetin %		Naringenin %		total flavone%	
		Before culture	After culture	Before culture	After culture	Before culture	After culture	Before culture	After culture	Before culture	After culture
Blank	None	0.19	0.19	5.89	5.89	0	0	0	0	9.11	9.11

Test group	*A. niger*	0.19	0.07	5.89	5.11	0	0.77	0	0.11	9.11	14.20
	*A. flavus*	0.19	0.19	5.89	5.69	0	0.30	0	0	9.11	9.78
	*P. minioluteum*	0.19	0.19	5.89	5.89	0	0	0	0	9.11	9.44
	*P. commune*	0.19	0.19	5.89	5.89	0	0	0	0	9.11	9.42
	*P. citrinum*	0.19	0.19	5.89	5.89	0	0	0	0	9.11	9.35

**Table 5:** The effect of SOD and MDA.

Group	Dose (g·kg^−1^)	SOD vitality (U/ml)	MDA vitality (U/ml)
Blank	0	648.04±9.19*∗∗*	196.5±2.42*∗*
CCl_4_ group	0	616.86±9.67	278.1±1.70
S1 group	16	640.85±9.57*∗∗*	215.4±2.49*∗*
One year PCR group	16	625.81±12.15	276.4±2.40
Three years PCR group	16	633.76±8.70*∗*	210.2±1.44*∗*

All values are means±SD (10-12). *∗*P<0.05 is considered statistically significant.

## Data Availability

The data used to support the findings of this study are included within the article, and all the data are available from the corresponding author upon request. The email address is liuyouping@163.com.
